# Supplementary material for: Development of a core outcome set for multimorbidity trials in low/middle-income countries (COSMOS): study protocol
Source: BMJ Open. 2022 Feb 15;12(2):e051810. doi: 10.1136/bmjopen-2021-051810 (PMC8852662; doi:10.1136/bmjopen-2021-051810)
Supplement: Author's manuscript [file bmjopen-2021-051810.draft_revisions.pdf]

BMJ Open is committed to open peer review. As part of this commitment we make the peer review history of every article we publish publicly available.

When an article is published we post the peer reviewers' comments and the authors' responses online. We also post the versions of the paper that were used during peer review. These are the versions that the peer review comments apply to.

The versions of the paper that follow are the versions that were submitted during the peer review process. They are not the versions of record or the final published versions. They should not be cited or distributed as the published version of this manuscript.

BMJ Open is an open access journal and the full, final, typeset and author-corrected version of record of the manuscript is available on our site with no access controls, subscription charges or pay-per-view fees (http://bmjopen.bmj.com).

If you have any questions on BMJ Open's open peer review process please email info.bmjopen@bmj.com

BMJ Open

Development of a core outcome set for multimorbidity trials in low- and middle-income countries (COSMOS): Study Protocol

Journal:	*BMJ Open*
Manuscript ID	bmjopen-2021-051810
Article Type:	Protocol
Date Submitted by the Author:	30-Mar-2021

Complete List of Authors:	Boehnke, Jan Rasmus<br>Rana, Rusham Zahra<br>Kirkham, Jamie J.<br>Rose, Louise<br>Agarwal, Gina<br>Barbui, Corrado<br>Chase, Alyssa<br>Churchill, Rachel<br>Flores-Flores, Oscar<br>Hurst, John<br>Levitt, Naomi<br>van Olmen, Josefien<br>Purgato, Marianna<br>Siddiqi, Kamran<br>Uphoff, Eleonora<br>Vedanthan, Rajesh<br>Wright, Judy<br>Wright, Kath<br>Zavala, Gerardo A.<br>Siddiqi, Najma

Keywords:	Clinical trials < THERAPEUTICS, STATISTICS & RESEARCH METHODS, GENERAL MEDICINE (see Internal Medicine)

SCHOLARONE™
Manuscripts

Development of a core outcome set for multimorbidity trials in low- and middle-income countries (COSMOS): Study Protocol

Jan R. Boehnke[1,2], Rusham Zahra Rana[3], Jamie J. Kirkham[4], Louise Rose[5], Gina Agarwal[6], Corrardo Barbui[7,8], Alyssa Chase[9], Rachel Churchill[10], Oscar Flores-Flores[11,12], John R. Hurst[13], Naomi Levitt[14], Josefien van Olmen[15], Marianna Purgato[7,8], Kamran Siddiqi[2,16], Eleonora Uphoff[10], Rajesh Vedanthan[17], Judy Wright[18], Kath Wright[19], Gerardo A. Zavala[2], Najma Siddiqi[2,16]

Affiliations:

1. School of Health Sciences, University of Dundee, United Kingdom.
2. Department of Health Sciences, University of York, York, United Kingdom.
3. Institute of Psychiatry, Rawalpindi Medical University, Pakistan.
4. Centre for Biostatistics, Manchester Academic Health Science Centre, University of Manchester, Manchester, United Kingdom.
5. Florence Nightingale Faculty of Nursing, Midwifery and Palliative Care, King's College London, London, United Kingdom.
6. Department of Family Medicine, McMaster University, Hamilton, Canada.
7. WHO Collaborating Centre for Research and Training in Mental Health and Service Evaluation Department of Neurosciences, Biomedicine and Movement Sciences, Section of Psychiatry, University of Verona, Verona, Italy.
8. Cochrane Global Mental Health, University of Verona, Verona, Italy.
9. Global Alliance for Chronic Diseases, United Kingdom.
10. Cochrane Common Mental Disorders and Centre for Reviews and Dissemination, University of York, United Kingdom.
11. Facultad de Medicina Humana, Centro de Investigación del Envejecimiento (CIEN), Universidad de San Martin de Porres, Lima, Peru.
12. Asociación Benéfica PRISMA, Lima, Peru.
13. UCL Respiratory, University College London, United Kingdom.
14. Division of Diabetic Medicine and Endocrinology, Groote Schuur Hospital and University of Cape Town, South Africa.

For peer review only - http://bmjopen.bmj.com/site/about/guidelines.xhtml

15. University of Antwerp, Department of Family Medicine and Population Health, Belgium.

16. Hull York Medical School, York, United Kingdom.

17. Department of Population Health, NYU Grossman School of Medicine, New York University, New York, NY, United States of America.

18. Leeds Institute of Health Sciences, University of Leeds, United Kingdom.

19. Centre for Reviews and Dissemination, University of York, United Kingdom.

Corresponding author:

Jan R. Boehnke

0000-0003-0249-1870

j.r.boehnke@dundee.ac.uk

School of Health Sciences

University of Dundee

Dundee, DD1 4HJ

United Kingdom

For peer review only

Author contribution statement:

Najma Siddiqi and Jan Boehnke conceived the study. Jan Boehnke, Jamie Kirkham, Rusham Rana, Louise Rose and Najma Siddiqi led the design of the research. Jan Boehnke, Najma Siddiqi and Rusham Rana drafted the manuscript. All authors contributed to critiquing and revising the manuscript for important intellectual content.

Declaration of funding and other support:

This research is supported in part by the National Institute for Health Research (NIHR) using Official Development Assistance (ODA) funding (Grant: 17/63/130: NIHR Global Health Research Group: Improving Outcomes in Mental and Physical Multimorbidity and Developing Research Capacity (IMPACT) in South Asia at the University of York).

For peer review only - http://bmjopen.bmj.com/site/about/guidelines.xhtml

The research is also receiving support from Bradford District Care NHS Foundation Trust and a NIHR Global Health Research Short Placement Award for Research Collaboration to Rusham Zahra Rana (SPARC PILOT 01-19-03). Oscar Flores-Flores is supported by the Research Training in Chronic, non-communicable respiratory diseases in Peru, Fogarty International Center, US National Institutes of Health (D43TW011502).

The core outcome set development is also supported by the Global Alliance for Chronic Diseases (GACD) Multimorbidity Working Group; the World Psychiatry Association; and by Cochrane Common Mental Disorders which is funded by NIHR (NIHR 129457) and Cochrane Global Mental Health (https://globalmentalhealth.cochrane.org). It is supported by the NCD Alliance by dissemination of participation opportunities to people living with multiple health conditions in its network.

Acknowledgements:

The authors thank Job van Boven (University Medical Center Groningen, University of Groningen, The Netherlands) and Meena Daivadanam (Department of Food Studies, Nutrition and Dietetics, Uppsala University, Sweden) for comments on earlier drafts of this protocol.

For peer review only - http://bmjopen.bmj.com/site/about/guidelines.xhtml

Abstract

Introduction

'Multimorbidity' describes the presence of two or more long-term conditions, which can include communicable and non-communicable diseases, and mental disorders. The rising global burden from multimorbidity is well-documented, but trial evidence for effective interventions in low- and middle-income countries (LMICs) is limited. Selection of appropriate outcomes is fundamental to trial design to ensure cross-study comparability, but there is currently no agreement on a core outcome set (COS) to include in trials investigating multimorbidity specifically in LMIC. Our aim is to develop international consensus on two COS for trials of interventions to prevent and treat multimorbidity in LMIC settings.

Methods and Analysis

Following methods recommended by the Core Outcome Measures in Effectiveness Trials (COMET initiative), the development of these two COS will occur in three stages: (1) generation of a long list of potential outcomes for inclusion; (2) two-round online Delphi surveys; and (3) consensus meetings. First, to generate an initial list of outcomes, we will conduct a systematic review of multimorbidity intervention and prevention trials and interviews with people living with multimorbidity and their caregivers in LMICs. Outcomes will be classified using an outcome taxonomy. Two-round Delphi surveys will be used to elicit importance scores for these outcomes from people living with multimorbidity, caregivers, healthcare professionals, policy makers, and researchers in LMICs. Finally, consensus meetings will be held to discuss the Delphi survey results and agree outcomes for inclusion in the two COS.

Ethics and dissemination

The study has been approved by the Research Governance Committee of the Department of Health Sciences, University of York, UK [HSRGC/2020/409/D:COSMOS]. Each participating country/research group will obtain local ethics board approval. Informed consent will be obtained from all participants. We will disseminate findings through peer-reviewed open access publications, and presentations at global conferences selected to reach a wide range of LMIC stakeholders.

For peer review only - http://bmjopen.bmj.com/site/about/guidelines.xhtml

Trial registration:

PROSPERO ID CRD42020197293

COMET https://www.comet-initiative.org/Studies/Details/1580

Keywords: Core Outcome Set, Comorbidity, Multimorbidity, Low- and Middle-Income Countries, Delphi, Consensus

Strengths and limitations of this study

- The development process follows guidelines and best practice recommendations for developing core outcome sets and integrates four sources of information.
- Interviews with people living with multimorbidity and caregivers are conducted in several LMICs (in South Asia, Africa and Latin America), by local teams, and in local languages to identify outcomes relevant to them.
- The Delphi survey and consensus meetings are conducted in English which limits the breadth of participation in these stages of the process.
- Despite involvement of a wide range of LMIC stakeholders in the process, there may be some limitations to the generalisability of the final core outcome sets due to the heterogeneity of target conditions and the diversity of countries, cultures and experiences.

For peer review only - http://bmjopen.bmj.com/site/about/guidelines.xhtml

INTRODUCTION

The rising global burden from chronic diseases is widely recognised and its detrimental impact on individuals as well as on societies is well-documented(1,2). Chronic diseases often co-occur in individuals and this 'multimorbidity' is associated with more frequent health care consultations(3), longer hospital stays(4), worse health-related quality of life(5–7), increased health care costs(8), higher out-of-pocket expenditure for health care(8), and higher mortality(9). These factors make multimorbidity a pressing challenge for health care systems as well as for patients, families and caregivers(2,8,10). We define multimorbidity as the presence of two or more chronic (or long term) health conditions(11) encompassing communicable and non-communicable diseases and mental disorders e.g. depression and diabetes occurring together. Chronic communicable diseases are those that affect people over a number of months or years e.g. HIV or tuberculosis.

Estimates of the prevalence of multimorbidity in high-income countries (HIC) vary from 25% to 60% in health care and community settings(12–14). The prevalence of multimorbidity in low- and middle-income countries (LMICs) appears to be lower(15,16) but is set to rise rapidly due to demographic and lifestyle changes. A Cochrane review on the effectiveness of health service or patient-oriented interventions to improve outcomes in people with multimorbidity in primary care and community settings found most evidence was from HICs(17). This evidence cannot readily be applied to LMICs where differing patterns of disease, health care systems, resources and cultural considerations will affect the acceptability and effectiveness of interventions. Trials of interventions to tackle multimorbidity specifically in LMICs are urgently needed.

To generate a robust evidence-base, results from individual trials will need to be compared and synthesised. This requires studies to use a 'core outcome set' (COS), i.e. an agreed minimum set of outcomes to be measured in all trials of a specific intervention for a health condition(18). A COS for multimorbidity research has been previously developed(19); it includes three outcomes: health-related quality of life, mental health, and mortality. However, a limitation of this COS is that it was largely based on the evidence and expert opinions from HICs(20,21), with no involvement of LMIC stakeholders in reaching consensus. A recent review discussed outcome measures across eight domains that may be suitable for multimorbidity research in LMICs. However, it did not systematically review the literature, patients and caregivers were not involved, and outcomes were not prioritised using established consensus methods.(22) The authors concluded that development of a COS for LMICs would be an important contribution to the field.

Whilst some research outcomes may be applicable to both LMICs and HICs, there are important differences in disease patterns and health care systems that need to be considered. In a recent trials

For peer review only - http://bmjopen.bmj.com/site/about/guidelines.xhtml

methodology research priority setting exercise for LMIC settings, the choice of outcomes to measure was identified as the number one most important topic (23). This protocol outlines a plan to bridge the gap and develop two COS for research on the prevention and treatment of multimorbidity in an LMIC setting, using the minimum standards approach for developing COS (24).

Aims and objectives

We aim to develop two COS for trials of interventions designed to (1) prevent and (2) treat multimorbidity in LMIC settings. The scope includes all types of interventions for multimorbidity – pharmacological, non-pharmacological, simple and complex in adults (aged 18 years and over) at risk of, or living with multimorbidity, in community, primary care and hospital settings in LMIC.

METHODS

We will follow approaches recommended by the Core Outcome Measures in Effectiveness Trials (COMET) initiative(25) and Outcome Measures in Rheumatoid Arthritis Clinical Trials (OMERACT(26)). The COS development has been registered with COMET (https://www.comet-initiative.org/Studies/Details/1580). The process will follow the minimum standards for the design of a COS study (COS-STAD(24)), which includes the careful consideration of the scope, stakeholders, and the consensus process. This protocol is structured according to the COS-STAP statement(27).

Our research team has broad global LMIC representation as well as experience of working in LMICs. The COSMOS working group supporting the study draws on a network of 38 research teams in LMICs that will be actively involved in different stages of COS development. We will ensure patient, community and public involvement throughout the development and delivery of the project, and include people with lived experience and their caregivers as members in the core research team.

COS development will involve three stages: (1) generation of a long list of outcomes, (2) two-round online Delphi consensus-building surveys and (3) online consensus meetings.

1. Outcome list generation

A long-list of outcomes will be generated from two sources: first, a systematic review of outcomes reported in trials of interventions conducted in LMICs for the prevention and treatment of multimorbidity, and second, using interviews with people living with multimorbidity and their caregivers from LMICs.

For peer review only - http://bmjopen.bmj.com/site/about/guidelines.xhtml

(1A) Systematic review

We will conduct a systematic review to identify an initial list of potential outcomes from trials of multimorbidity interventions conducted in adults living in LMICs. The systematic review protocol has been registered, and full details are available on PROSPERO (CRD42020197293).

Search methods:

We will search electronic databases (e.g. MEDLINE, Embase, PsycINFO) from 1990 to present. We will also search for relevant systematic reviews in The Cochrane Library, the Database of Abstracts of Reviews of Effects (DARE), PROSPERO and Joanna Briggs; and unpublished ongoing trials in ClinicalTrials.gov, ISRCTN registry and in the International Clinical Trials Registry Platform (http://apps.who.int/trialsearch). We will apply a study design filter to identify interventional studies. We will not limit inclusion by language.

Selection criteria

We will include randomised (individual, cluster, and cross-over randomisation) intervention studies published in either protocols or definitive reports. Studies of adults aged 18 years and above, living in low- and middle-income countries, with multimorbidity, or at risk of multimorbidity will be eligible. Low- and middle-income countries will be defined using the 2019 World Bank definitions. Studies of all interventions, whether pharmacological, non-pharmacological, simple or complex, for the prevention or treatment of multimorbidity (or both) and any comparators will be included.

Study selection

Two reviewers will independently screen titles/ abstracts with any discrepancies resolved through consensus or if needed, by a third reviewer. Full texts will also be reviewed following a similar process.

Data collection

Data will be extracted from eligible studies on publication date, years when the trial was conducted, country, clinical setting (e.g., primary care, specialised services), type of intervention, target conditions, and outcomes, by a single reviewer, with 10% of extraction records also reviewed by a second senior reviewer.

Quality assessment

Risk of bias will not be assessed, as the purpose is only to assemble a list of outcomes that have been used in previous studies.

Data synthesis

Outcomes will be grouped based on whether they were used to evaluate prevention or treatment of multimorbidity. For both groups of outcomes, we will construct matrices to identify the outcomes,

For peer review only - http://bmjopen.bmj.com/site/about/guidelines.xhtml

whether primary or secondary, their definitions and measures used in each study. We will tabulate the proportion of included studies that report on each outcome (incl. primary versus other outcome) and rank-order the outcomes accordingly. In a second step, to organise the outcomes we will use an outcome taxonomy.

The outcomes will be presented by intervention type (prevention, treatment), by combination of conditions and by region of the world (World Bank regions of LMICs) in which the study was conducted.

(1B) Interviews

To identify outcomes of importance to people from LMICs living with multimorbidity and their families or caregivers, we will conduct semi-structured interviews. Participants will be approached by local research teams either in-person or via phone, post or on-line (based on the local research site resources and COVID-19 restrictions).

Inclusion criteria for interviews comprise: people living with (or caring for someone living with) two or more health conditions; at least 18 years of age; being able to visit the local partner organisation's site or access to telephone/Internet; and consenting to participate. Participants will be identified through contact with relevant health care services, support and advocacy groups, charities and use of social media including Twitter, adverts placed on public and patient involvement websites, snowballing techniques, and personal contacts. We will use purposive sampling (28) based on the following characteristics: age (over/under 65 years); sex (male/female); using community or primary care versus secondary/specialist healthcare services; and region (World Bank income groups: low, lower middle, upper middle(29)). We will include a range of LMICs in diverse geographic locations. We anticipate 15 to 20 participants from each group (45-60 interviews), which should be sufficient to achieve diversity of viewpoints and data saturation(30).

We will collect demographic information (age, sex, marital status, highest level of education, socioeconomic status, occupation and disability. We have developed a semi-structured interview guide (see Appendix 1(31)), which will be translated and back-translated by local teams in the local language. All interviews will be audio recorded or if not possible (because of technology limitations or the participant withholding consent) will be recorded in contemporaneous notes.

The local research teams will be asked to transcribe and translate into English two sections of the interview, (i) participants' consent and (ii) content relevant to study outcomes. Interview transcripts will be reviewed to confirm health outcomes to put forward for the Delphi.

For peer review only - http://bmjopen.bmj.com/site/about/guidelines.xhtml

Outcomes identified in the interviews will be integrated with findings from the systematic review. All unique outcomes identified from the systematic review and interviews will be categorised into domains for presentation in the Delphi survey using Dodd's outcome taxonomy(32).

2. Delphi surveys with stakeholders

We will conduct separate Delphi surveys for the prevention and treatment COS; however, survey participants will be common to both.

We will recruit a range of stakeholders living or working in LMICs including the following groups: i) people living with multimorbidity and their family caregivers, ii) healthcare professionals; iii) policy makers and managers; and iv) researchers interested in multimorbidity with experience in LMIC contexts. Participants will be identified using connected networks (e.g. NIHR Global Health Research Groups/Units, Global Alliance for Chronic Diseases, World Psychiatric Association, NCD Alliance) and our project network across 38 countries to distribute information about the study, including professional societies and non-government organisations relevant to multimorbidity and government ministries. For recruitment to the researcher stakeholder group, we will send personalised emails to corresponding authors living or working in LMICs identified via our systematic review and request them to snowball to co-authors and other relevant contacts.

We will aim to maintain a minimum of approximately 20 participants representing each stakeholder group (total approximately 80 participants) throughout Delphi rounds, with representation across the World Bank LMIC income groups. We will oversample for the first round based on an estimated attrition of 30% across rounds. In both rounds we will send three reminders across three weeks to participants to complete the surveys and keep the surveys open for longer if needed to reduce attrition and improve the response rate.

(2A) Round one

We will include all outcomes identified through our systematic review and interviews, seeking advice from our community and public representatives to describe outcomes in lay terms. We will use an online survey tool (DelphiManager) to administer the surveys. We will pilot the questionnaire with eight individuals (two from each stakeholder group) to assess content validity and understanding.

We will list outcomes in domains according to the taxonomy classification. Since we are interested in assessing the importance of each outcome, we will ask participants to score each outcome (separately for prevention and for treatment) on a scale from 1 to 9 (1 to 3 = not important for inclusion; 4 to 6 = important but not critical; 7 to 9 = critical for inclusion(33)), considering

For peer review only - http://bmjopen.bmj.com/site/about/guidelines.xhtml

relevance for and feasibility of the outcome in LMIC settings. There will also be an option for 'unable to score'. We will provide the opportunity for participants to add additional outcomes and other comments. We will collect demographic information to describe our study sample (Table 1).

INSERT TABLE 1 ABOUT HERE

(2B) Round two

The research team will review any additional outcomes suggested in round one for inclusion. Any newly suggested outcomes will be taken forward if they are (i) relevant to the scope of the COS and (ii) not yet represented in the list of outcomes. All outcomes from round 1 and any new outcomes will be included in round 2.

In round two, we will provide participants with their own round one response; summarised responses of the whole group; and responses summarised by each stakeholder group. We will ask them to re-score the importance of each outcome in light of the extra information received, again separately for prevention and treatment. New outcomes from round one will be provided for scoring on the same 1 to 9 importance scale.

We will summarise round 2 responses separately for prevention and treatment using descriptive statistics, noting for each outcome the number and percentage of responses in each of the following categories:

- 1 to 3 = not important for inclusion
- 4 to 6 = important but not critical
- 7 to 9 = critical for inclusion

We will categorise outcomes according to the predefined consensus definition described in Table 2(34–36). All responses, including from partially completed questionnaires will be used in analyses.

We will also examine the percentage distribution of scores for each outcome by stakeholder group, geographic region and World Bank income group and present these graphically for the consensus meeting.

For peer review only - http://bmjopen.bmj.com/site/about/guidelines.xhtml

At the end of the second round of Delphi surveys participants will be sent information about the consensus meeting and requested to contact the team if they are interested in participating.

3. Outcome consensus meetings

Due to the wide geographic distribution of participants, the final step in the consensus process will be two online consensus meetings: one to agree the COS for prevention of multimorbidity and one for the COS for treatment of multimorbidity.

Participants of the Delphi surveys who complete both rounds and who express an interest in attending the consensus meeting will be purposively selected to ensure similar numbers from each stakeholder group, geographical spread and representation from target World Bank income groups. The meetings will be chaired by a facilitator, experienced in COS development. Results of the two-round Delphi surveys will be presented. Outcomes that have reached consensus 'in' or 'no consensus' (see Table 2) will be discussed further.

We will use a modified nominal group technique to achieve consensus. This will involve iterative small and whole group discussions and ranking of outcomes in order to agree which should be included in the two COS. To avoid duplication or overlap, the selected outcomes will also be discussed to ensure each relates to a distinct construct.

For people living with multimorbidity and their caregivers, we will provide additional support before the consensus meetings to explain the process and enable them to participate more fully in the discussions and ranking.

INSERT TABLE 2 ABOUT HERE

PATIENT AND PUBLIC INVOLVEMENT

There were no people living with the condition nor their caregivers involved in the planning of the study. Both will be involved in the project management group overseeing the Delphi surveys and consensus meeting. Both will participate in both Delphi surveys and consensus meeting.

For peer review only - http://bmjopen.bmj.com/site/about/guidelines.xhtml

ETHICS

The Ethics committee of the University of York has approved the study [HSRGC/2020/409/D: COSMOS]. Approvals will also be sought from relevant local ethics committees for all participating sites.

We will obtain written consent from participants in interviews and consensus meetings (or where conducted online, verbal consent will be recorded). For the Delphi surveys, we will seek consent for participation as part of the online questionnaire and consent to store contact data for completion of the next Delphi round and for invitation to consensus meetings. We will emphasise that participation is voluntary, participants are free to withdraw at any stage, and survey responses will be anonymized.

Data management

Interview data as well as anonymized Delphi data after secure download will be stored on a secure and encrypted database on a secure password protected server (the University of York, Department of Health Sciences File store Area). The access, use and storage of sensitive or confidential data will be conducted in accordance with the University of York Data Security Policy and Handling Sensitive Data Guidance.

DISSEMINATION

We will disseminate our findings through peer-reviewed and open access publications, and presentations at global conferences, selected to reach a wide range of LMIC stakeholders including health professionals and researchers and taking into account geographic locations. We will also provide lay summaries including infographics in relevant languages for community and public audiences and share these with interview and Delphi participants. Such summaries and short briefing papers will be made available freely on the COSMOS web page (https://www.impactsouthasia.com/) and in formats which are at the moment being successfully developed and trialled in the associated research networks (Global Alliance for Chronic Diseases website, World Psychiatric Association). In the longer term, success of dissemination will be assessed by how frequently the Multimorbidity COS is used in trials and cited in manuscripts(27).

DISCUSSION

We aim to develop and report two COS for multimorbidity outcomes specifically for LMIC by identifying outcomes that have been reported in the literature, including the views of people living with multimorbidity and their caregivers through interviews, and developing consensus using Delphi surveys and consensus meetings. Development of the two COS aims to increase the

For peer review only - http://bmjopen.bmj.com/site/about/guidelines.xhtml

consistency of selection, collection and reporting of outcomes in future studies of interventions to prevent and to treat multimorbidity.

A considerable strength of the study is the involvement of people living with multimorbidity and their family caregivers throughout, including in interviews to gain their perspective on important outcomes. This involvement is increasingly recognised as an important contributor to COS (24,37,38) as it highlights new outcomes from the service user perspective. Aiming for approximately fifty participants from interviews and a wide range of participants in the Delphi globally(39), the study will generate outcomes and gain consensus from a range of diverse settings.

The research team comprises experienced researchers and experts in the field of multimorbidity and COS development. The support of the Global Alliance for Chronic Disease (GACD), the World Psychiatry Association (WPA) and partners in IMPACT (Improving Outcomes in Mental and Physical Multimorbidity and Developing Research Capacity) will help provide links to local groups and identify appropriate stakeholders for the consensus process as well as for the dissemination of the outcomes generated from the study.

There are some relevant limitations. First, the scope is broad; despite the systematic effort to conduct a comprehensive and inclusive COS development, with involvement of a wide range of LMIC stakeholders, it is possible that there may be limitations to the generalisability of the final core outcome sets due to heterogeneity of interventions, target conditions and diversity of countries, cultures and experiences.

A further limitation is that although the study aims to develop COS that will be relevant to LMICs more generally, it will only include representation from some LMIC regions. We recognise that LMICs are not a homogenous category and social and cultural differences will necessitate future research investigating how these COS can be applied to other regions.

To be flexible and adaptable for a range of LMIC settings, and feasible within the available time and resource constraints, the planned interviews are limited in number and relatively short and semi-structured and the transcription will focus only on the most relevant sections. Due to internationally different clinical standards and the potential stigma associated with labelling people as "at risk", we decided not to conduct interviews focusing on prevention only. This may risk missing outcomes which would be obtained from a more in-depth exploration

Although our aim is to secure participation from a wide spread of geographical areas, there are a number of challenges to involvement. To take part in Delphi surveys and consensus meetings, English proficiency, a computer and Internet access will be required. This poses challenges for engagement with some stakeholder groups, inclusiveness and participant attrition. Additionally,

For peer review only - http://bmjopen.bmj.com/site/about/guidelines.xhtml

since patient and public involvement is still a developing concept in many contexts and specifically in global health research(40–43) identifying appropriate patient representatives for our consensus panel may prove challenging.

Despite these challenges, this is the first attempt to develop core outcome sets for multimorbidity seeking input from a wide range of LMIC partners and participants(21).

Our COSMOS study follows a systematic widely practiced approach to generate core outcomes and meets the methodological standards put forth by the COMET initiative. It will generate a template for steps in developing international consensus involving LMICs, and two COS for use in future multimorbidity prevention and treatment trials, helping to generate evidence to address this important global health priority.

References

1. Murray CJL, Barber RM, Foreman KJ, Ozgoren AA, Abd-Allah F, Abera SF, et al. Global, regional, and national disability-adjusted life years (DALYs) for 306 diseases and injuries and healthy life expectancy (HALE) for 188 countries, 1990–2013: quantifying the epidemiological transition. The Lancet. 2015 Nov 28;386(10009):2145–91.

2. Pati S, Swain S, Knottnerus JA, Metsemakers JFM, van den Akker M. Magnitude and determinants of multimorbidity and health care utilization among patients attending public versus private primary care: a cross-sectional study from Odisha, India. Int J Equity Health. 2020 29;19(1):57.

3. Palladino R, Tayu Lee J, Ashworth M, Triassi M, Millett C. Associations between multimorbidity, healthcare utilisation and health status: evidence from 16 European countries. Age Ageing. 2016 May;45(3):431–5.

4. Mai Q, Holman CDJ, Sanfilippo FM, Emery JD. The impact of mental illness on potentially preventable hospitalisations: a population-based cohort study. BMC Psychiatry. 2011 Oct 10;11(1):163.

5. Barra M, Augestad LA, Whitehurst DGT, Rand-Hendriksen K. Examining the relationship between health-related quality of life and increasing numbers of diagnoses. Qual Life Res Int J Qual Life Asp Treat Care Rehabil. 2015 Dec;24(12):2823–32.

6. Ou H-T, Lin C-Y, Erickson SR, Balkrishnan R. Refined comorbidity index based on dimensionality of comorbidity for use in studies of health-related quality of life. Qual Life Res Int J Qual Life Asp Treat Care Rehabil. 2016;25(10):2543–57.

7. Tyack Z, Frakes K-A, Barnett A, Cornwell P, Kuys S, McPhail S. Predictors of health-related quality of life in people with a complex chronic disease including multimorbidity: a longitudinal cohort study. Qual Life Res Int J Qual Life Asp Treat Care Rehabil. 2016;25(10):2579–92.

For peer review only - http://bmjopen.bmj.com/site/about/guidelines.xhtml

8. Zhao Y, Atun R, Oldenburg B, McPake B, Tang S, Mercer SW, et al. Physical multimorbidity, health service use, and catastrophic health expenditure by socioeconomic groups in China: an analysis of population-based panel data. Lancet Glob Health. 2020 Jun 1;8(6):e840–9.

9. González R, López-Herce J, González R, López-Herce J. Mortality of patients with chronic disease: an increasing problem,. J Pediatr (Rio J). 2019 Dec;95(6):625–7.

10. Hurst JR, Dickhaus J, Maulik PK, Miranda JJ, Pastakia SD, Soriano JB, et al. Global Alliance for Chronic Disease researchers' statement on multimorbidity. Lancet Glob Health. 2018 Dec 1;6(12):e1270–1.

11. The Academy of Medical Sciences. Multimorbidity: a priority for global health research [Internet]. 2018 Apr [cited 2021 Jan 7] p. 127. Available from: https://acmedsci.ac.uk/file-download/82222577

12. Brett T, Arnold-Reed DE, Popescu A, Soliman B, Bulsara MK, Fine H, et al. Multimorbidity in Patients Attending 2 Australian Primary Care Practices. Ann Fam Med. 2013 Nov;11(6):535–42.

13. Marengoni A, Winblad B, Karp A, Fratiglioni L. Prevalence of chronic diseases and multimorbidity among the elderly population in Sweden. Am J Public Health. 2008 Jul;98(7):1198–200.

14. Schäfer I, Hansen H, Schön G, Höfels S, Altiner A, Dahlhaus A, et al. The influence of age, gender and socio-economic status on multimorbidity patterns in primary care. First results from the multicare cohort study. BMC Health Serv Res. 2012 Apr 3;12:89.

15. Afshar S, Roderick PJ, Kowal P, Dimitrov BD, Hill AG. Global Patterns of Multimorbidity: A Comparison of 28 Countries Using the World Health Surveys. Appl Demogr Public Health 21st Century. 2017;381–402.

16. Xu X, Mishra GD, Jones M. Mapping the global research landscape and knowledge gaps on multimorbidity: a bibliometric study. J Glob Health. 2017 Jun;7(1):010414.

17. Smith SM, Wallace E, O'Dowd T, Fortin M. Interventions for improving outcomes in patients with multimorbidity in primary care and community settings. Cochrane Database Syst Rev [Internet]. 2016 [cited 2020 Nov 26];(3). Available from: https://www.cochranelibrary.com/cdsr/doi/10.1002/14651858.CD006560.pub3/full

18. Prinsen CAC, Vohra S, Rose MR, Boers M, Tugwell P, Clarke M, et al. How to select outcome measurement instruments for outcomes included in a "Core Outcome Set" – a practical guideline. Trials. 2016 Sep 13;17(1):449.

19. Smith SM, Wallace E, Salisbury C, Sasseville M, Bayliss E, Fortin M. A Core Outcome Set for Multimorbidity Research (COSmm). Ann Fam Med. 2018 Mar 1;16(2):132–8.

20. Hailemariam M, Pathare S. The missing global in global mental health. Lancet Psychiatry. 2020 Dec 1;7(12):1011–2.

For peer review only

For peer review only - http://bmjopen.bmj.com/site/about/guidelines.xhtml

21. Lee A, Davies A, Young AE. Systematic review of international Delphi surveys for core outcome set development: representation of international patients. BMJ Open. 2020 Nov 1;10(11):e040223.

22. Hurst JR, Agarwal G, van Boven JFM, Daivadanam M, Gould GS, Wan-Chun Huang E, et al. Critical review of multimorbidity outcome measures suitable for low-income and middle-income country settings: perspectives from the Global Alliance for Chronic Diseases (GACD) researchers. BMJ Open. 2020 Sep;10(9):e037079.

23. Rosala-Hallas A, Bhangu A, Blazeby J, Bowman L, Clarke M, Lang T, et al. Global health trials methodological research agenda: results from a priority setting exercise. Trials. 2018 Feb 5;19(1):48.

24. Kirkham JJ, Davis K, Altman DG, Blazeby JM, Clarke M, Tunis S, et al. Core Outcome Set-STAndards for Development: The COS-STAD recommendations. PLoS Med. 2017 Nov;14(11):e1002447.

25. Williamson PR, Altman DG, Bagley H, Barnes KL, Blazeby JM, Brookes ST, et al. The COMET Handbook: version 1.0. Trials. 2017 Jun 20;18(Suppl 3):280.

26. OMERACT Handbook [Internet]. OMERACT Handbook. 2018 [cited 2021 Jan 7]. Available from: https://omeracthandbook.org/

27. Kirkham JJ, Gorst S, Altman DG, Blazeby JM, Clarke M, Tunis S, et al. Core Outcome Set-STAndardised Protocol Items: the COS-STAP Statement. Trials. 2019 Feb 11;20(1):116.

28. Pope C, Mays N. Qualitative Research: Reaching the parts other methods cannot reach: an introduction to qualitative methods in health and health services research. BMJ. 1995 Jul 1;311(6996):42–5.

29. World Bank Country and Lending Groups [Internet]. The World Bank. [cited 2021 Jan 7]. Available from: https://datahelpdesk.worldbank.org/knowledgebase/articles/906519-world-bank-country-and-lending-groups

30. Francis JJ, Johnston M, Robertson C, Glidewell L, Entwistle V, Eccles MP, et al. What is an adequate sample size? Operationalising data saturation for theory-based interview studies. Psychol Health. 2010 Dec;25(10):1229–45.

31. Williamson PR, Altman DG, Bagley H, Barnes KL, Blazeby JM, Brookes ST, et al. The COMET Handbook: version 1.0. Trials. 2017 Jun 20;18(Suppl 3):280.

32. Dodd S, Clarke M, Becker L, Mavergames C, Fish R, Williamson PR. A taxonomy has been developed for outcomes in medical research to help improve knowledge discovery. J Clin Epidemiol. 2018;96:84–92.

33. Guyatt GH, Oxman AD, Kunz R, Atkins D, Brozek J, Vist G, et al. GRADE guidelines: 2. Framing the question and deciding on important outcomes. J Clin Epidemiol. 2011 Apr 1;64(4):395–400.

34. Harman NL, Wilding J, Curry D, Harris J, Logue J, Pemberton RJ, et al. Selecting Core Outcomes for Randomised Effectiveness trials In Type 2 Diabetes (SCORE-IT): study protocol for the development of a core outcome set. Trials. 2018 Aug 7;19(1):427.

For peer review only - http://bmjopen.bmj.com/site/about/guidelines.xhtml

35. Quirke FA, Healy P, Bhraonáin EN, Daly M, Biesty L, Hurley T, et al. COHESION: core outcomes in neonatal encephalopathy (protocol). Trials. 2021 Dec;22(1):125.

36. Williamson PR, Altman DG, Blazeby JM, Clarke M, Devane D, Gargon E, et al. Developing core outcome sets for clinical trials: issues to consider. Trials. 2012 Aug 6;13(1):132.

37. Jones JE, Jones LL, Keeley TJH, Calvert MJ, Mathers J. A review of patient and carer participation and the use of qualitative research in the development of core outcome sets. PLOS ONE. 2017 Mar 16;12(3):e0172937.

38. Young B, Bagley H. Including patients in core outcome set development: issues to consider based on three workshops with around 100 international delegates. Res Involv Engagem. 2016 Jul 8;2(1):25.

39. Duffy JMN, AlAhwany H, Bhattacharya S, Collura B, Curtis C, Evers JLH, et al. Developing a core outcome set for future infertility research: an international consensus development study†‡. Hum Reprod. 2020 Dec 1;35(12):2725–34.

40. Bedwell C, Lavender T. Giving patients a voice: implementing patient and public involvement to strengthen research in sub-Saharan Africa. J Epidemiol Community Health. 2020 Apr 1;74(4):307–10.

41. Cook N, Siddiqi N, Twiddy M, Kenyon R. Patient and public involvement in health research in low and middle-income countries: a systematic review. BMJ Open. 2019 May 1;9(5):e026514.

42. Simkhada B, van Teijlingen E, Naheed A, Warren A, Green S. Importance of involving patients and public in health research in Bangladesh and Nepal. Int J Technol Assess Health Care. 2020 Nov 5;37:e10.

43. Beran D, Pesantes MA, Berghusen MC, Hennig BJ, Jacobi J, Lazo-Porras M, et al. Rethinking research processes to strengthen co-production in low and middle income countries. BMJ. 2021 Feb 16;372:m4785.

For peer review only - http://bmjopen.bmj.com/site/about/guidelines.xhtml

Table 1: Delphi Stakeholder Characteristics

Stakeholder group	Characteristic
People with multimorbidity/family members/caregivers	
	Age (≤65; >65)
	Sex (male; female)
	Multimorbidities
	Community/primary care, secondary care; specialist healthcare
	Level of formal education (<= 10 years/>10years)
	Region of residence
Health professionals	
	Profession (doctor, nurse, allied health, community workers)
	Specialist area relevant to multimorbidity
	Region of residence
Policy makers and managers	
	Region of residence
Researchers	
	Specialist area relevant to multimorbidity
	Region of residence

For peer review only - http://bmjopen.bmj.com/site/about/guidelines.xhtml

Table 2: Definition of consensus for outcomes in the Delphi surveys

Consensus IN	Consensus that outcome should be included in the core outcome set	70% or more participants scoring 7–9<br>AND<br>< 15% participants scoring 1–3
Consensus OUT	Consensus that outcome should not be included in the core outcomes set	50% or fewer participants scoring 7 to 9 in each stakeholder group
No consensus	Uncertainty about importance of outcome	Anything else

For peer review only

For peer review only - http://bmjopen.bmj.com/site/about/guidelines.xhtml

Online Appendix to Boehnke et al., Development of a core outcome set for multimorbidity trials in low- and middle-income countries (COSMOS): Study Protocol. medRXix.

Appendix A: Interview guide

Development of a core outcome set for multimorbidity studies in low- and middle-income countries (COSMOS)

Interview Guide for Patients

Version: 1.3
Date: 28-10-20

Demographics (age, gender, marital status, highest level of education, socioeconomic status, occupation and disability should be recorded at eligibility checking/recruitment) need to be collected on a separate 'demographics' sheet.

Introduction:

Our goal is to understand what outcomes are important to you in preventing or treating co-existing chronic illnesses. We also want to identify what terms you might prefer to use to describe these outcomes.

What is an outcome?

To help patients, their family, doctors and other health professionals make decisions about treatments, we need evidence about what works best. Treatments are developed and tested by researchers to make sure they work and are safe. To do this, researchers need to look at the effects those treatments have on patients.

Researchers do this by measuring an 'outcome'. For example, in a study of how well a new asthma treatment works, 'outcomes' might include: a measure of how fast the asthma attack gets better; how many times a person has to stay in hospital because of asthma; or how well a person can get on with their usual activities because of asthma.

What is a core outcome set?

If all studies of a particular health condition(s) use the same outcomes, they can all be compared and combined. When a set of main outcomes has been agreed for a health condition(s), it's called a 'core outcome set'. If studies use these core outcomes, we could bring together all the studies to get a better understanding of which treatments are best.

What do we need you to tell us?

Core outcomes have to be relevant to patients, family members as well as health professionals and researchers. Some outcomes may be more important to patients and families than to healthcare professionals but without asking we will not know this and they might not get included in the core outcome list.

For peer review only - http://bmjopen.bmj.com/site/about/guidelines.xhtml

[Interviewer, before proceeding to the interview please check (if not already done):

- whether the participant information sheet had been shared and discussed;
- whether consent was obtained and documented.]

Interview:

As a quick reminder, we are interested in your experience of living with [living with someone living with] two or more health conditions at the same time. This is what we mean when using the terms "co-existing" or "multimorbidity". With health conditions we mean mental disorders, non-communicable diseases, and/or communicable diseases. In this interview we are interested in your view on what matters to you as the result of treatment and/or care for your conditions.

1. Please can you tell me how long (approximately) you [your family member] have had these co-existing chronic illnesses?

2. Where have you received treatment for these?
Probes: In the community, primary care, hospital, tertiary centre.

3. (For family members only) What is your relationship to your family member with these conditions?

4. Please tell me a little bit about your (your family member's) experience living with these conditions
Probe: What matters to you the most about the impact of these conditions?

5. What do you (your family member) hope to gain through treatment or care?

6. Speaking from your experience, how do you know that a treatment for your conditions is working?

[Interviewer, to finish off this question, ask]: What would you say your priorities are?

For peer review only
For peer review only - http://bmjopen.bmj.com/site/about/guidelines.xhtml

7. Considering this experience, what would you want future studies of prevention and treatment to look at in order to make it better for others in the future?

8. Is there anything else you'd like to talk about in relation to research for treatments to prevent or treat these conditions?

For peer review only

Online Appendix to Boehnke et al., Development of a core outcome set for multimorbidity trials in low- and middle-income countries (COSMOS): Study Protocol. medRXix

For peer review only - http://bmjopen.bmj.com/site/about/guidelines.xhtml

TITLE/ABSTRACT			
Title	1a	Identify in the title that the paper describes the protocol for the planned development of a COS	Yes
Abstract	1b	Provide a structured abstract	Yes
INTRODUCTION			
Background and objectives	2a	Describe the background and explain the rationale for developing the COS, and identify the reasons why a COS is needed and the potential barriers to its implementation	Yes
	2b	Describe the specific objectives with reference to developing a COS	Yes
Scope	3a	Describe the health condition(s) and population(s) that will be covered by the COS	Yes
	3b	Describe the intervention(s) that will be covered by the COS	Yes
	3c	Describe the context of use for which the COS is to be applied	Yes
METHODS			
Stakeholders	4	Describe the stakeholder groups to be involved in the COS development process, the nature of and rationale for their involvement and also how the individuals will be identified; this should cover involvement both as members of the research team and as participants in the study	Yes
Information sources	5a	Describe the information sources that will be used to identify the list of outcomes. Outline the methods or reference other protocols/papers	Yes
	5b	Describe how outcomes may be dropped/combined, with reasons	Yes
Consensus process	6	Describe the plans for how the consensus process will be undertaken	Yes

For peer review only

Consensus definition	7a	Describe the consensus definition	Yes
	7b	Describe the procedure for determining how outcomes will be added/combined/dropped from consideration during the consensus process	Yes
ANALYSIS			
Outcome scoring/feedback	8	Describe how outcomes will be scored and summarised, describe how participants will receive feedback during the consensus process	Yes
Missing data	9	Describe how missing data will be handled during the consensus process	Yes
ETHICS and DISSEMINATION			
Ethics approval/informed consent	10	Describe any plans for obtaining research ethics committee/institutional review board approval in relation to the consensus process and describe how informed consent will be obtained (if relevant)	Yes
Dissemination	11	Describe any plans to communicate the results to study participants and COS users, inclusive of methods and timing of dissemination	Yes
ADMINISTRATIVE INFORMATION			
Funders	12	Describe sources of funding, role of funders	Yes
Conflicts of interest	13	Describe any potential conflicts of interest within the study team and how they will be managed	Yes

For peer review only

BMJ Open

Development of a core outcome set for multimorbidity trials in low- and middle-income countries (COSMOS): Study Protocol

Journal:	*BMJ Open*
Manuscript ID	bmjopen-2021-051810.R1
Article Type:	Protocol
Date Submitted by the Author:	12-Oct-2021

Complete List of Authors:	Boehnke, Jan Rasmus; University of Dundee, School of Health Sciences; University of York, Department of Health Sciences<br>Rana, Rusham Zahra; Rawalpindi Medical University, Institute of Psychiatry<br>Kirkham, Jamie J.; The University of Manchester, Centre for Biostatistics, Manchester Academic Health Science Centre<br>Rose, Louise; King's College London, Florence Nightingale Faculty of Nursing, Midwifery and Palliative Care<br>Agarwal, Gina; McMaster University, Department of Family Medicine<br>Barbui, Corrado; University of Verona, WHO Collaborating Centre for Research and Training in Mental Health and Service Evaluation Department of Neurosciences, Biomedicine and Movement Sciences, Section of Psychiatry; University of Verona, Cochrane Global Mental Health<br>Chase, Alyssa; Global Alliance for Chronic Diseases<br>Churchill, Rachel; University of York, Cochrane Common Mental Disorders and Centre for Reviews and Dissemination<br>Flores-Flores, Oscar; Universidad San Martin de Porres, Facultad de Medicina Humana, Centro de Investigación del Envejecimiento (CIEN); Asociación Benéfica PRISMA<br>Hurst, John; University College London, UCL Respiratory<br>Levitt, Naomi; Groote Schuur Hospital, Division of Diabetic Medicine and Endocrinology; University of Cape Town<br>van Olmen, Josefien; University of Antwerp, Department of Family Medicine and Population Health<br>Purgato, Marianna; University of Verona, WHO Collaborating Centre for Research and Training in Mental Health and Service Evaluation Department of Neurosciences, Biomedicine and Movement Sciences, Section of Psychiatry; University of Verona, Cochrane Global Mental Health<br>Siddiqi, Kamran; University of York, Department of Heath Sciences; Hull York Medical School<br>Uphoff, Eleonora; University of York, Cochrane Common Mental Disorders and Centre for Reviews and Dissemination<br>Vedanthan, Rajesh; New York University, Department of Population Health, NYU Grossman School of Medicine<br>Wright, Judy; University of Leeds, Leeds Institute of Health Sciences<br>Wright, Kath; University of York, Centre for Reviews and Dissemination<br>Zavala, Gerardo A.; University of York, Department of Health Sciences

	Siddiqi, Najma; University of York, Department of Health Sciences; Hull York Medical School
**Primary Subject Heading**:	Global health
Secondary Subject Heading:	Patient-centred medicine, Research methods
Keywords:	STATISTICS & RESEARCH METHODS, Protocols & guidelines < HEALTH SERVICES ADMINISTRATION & MANAGEMENT, Quality in health care < HEALTH SERVICES ADMINISTRATION & MANAGEMENT, Clinical trials < THERAPEUTICS

SCHOLARONE™
Manuscripts

Development of a core outcome set for multimorbidity trials in low- and middle-income countries (COSMOS): Study Protocol

Jan R. Boehnke[1,2], Rusham Zahra Rana[3], Jamie J. Kirkham[4], Louise Rose[5], Gina Agarwal[6], Corrado Barbui[7,8], Alyssa Chase[9], Rachel Churchill[10], Oscar Flores-Flores[11,12], John R. Hurst[13], Naomi Levitt[14], Josefien van Olmen[15], Marianna Purgato[7,8], Kamran Siddiqi[2,16], Eleonora Uphoff[10], Rajesh Vedanthan[17], Judy Wright[18], Kath Wright[19], Gerardo A. Zavala[2], Najma Siddiqi[2,16]

Affiliations:

1. School of Health Sciences, University of Dundee, United Kingdom.
2. Department of Health Sciences, University of York, York, United Kingdom.
3. Institute of Psychiatry, Rawalpindi Medical University, Pakistan.
4. Centre for Biostatistics, Manchester Academic Health Science Centre, University of Manchester, Manchester, United Kingdom.
5. Florence Nightingale Faculty of Nursing, Midwifery and Palliative Care, King's College London, London, United Kingdom.
6. Department of Family Medicine, McMaster University, Hamilton, Canada.
7. WHO Collaborating Centre for Research and Training in Mental Health and Service Evaluation Department of Neurosciences, Biomedicine and Movement Sciences, Section of Psychiatry, University of Verona, Verona, Italy.
8. Cochrane Global Mental Health, University of Verona, Verona, Italy.
9. Global Alliance for Chronic Diseases, United Kingdom.
10. Cochrane Common Mental Disorders and Centre for Reviews and Dissemination, University of York, United Kingdom.
11. Facultad de Medicina Humana, Centro de Investigación del Envejecimiento (CIEN), Universidad de San Martin de Porres, Lima, Peru.
12. Asociación Benéfica PRISMA, Lima, Peru.
13. UCL Respiratory, University College London, United Kingdom.
14. Division of Diabetic Medicine and Endocrinology, Groote Schuur Hospital and University of Cape Town, South Africa.

For peer review only

15. University of Antwerp, Department of Family Medicine and Population Health, Belgium.

16. Hull York Medical School, York, United Kingdom.

17. Department of Population Health, NYU Grossman School of Medicine, New York University, New York, NY, United States of America.

18. Leeds Institute of Health Sciences, University of Leeds, United Kingdom.

19. Centre for Reviews and Dissemination, University of York, United Kingdom.

Corresponding author:

Jan R. Boehnke

0000-0003-0249-1870

j.r.boehnke@dundee.ac.uk

School of Health Sciences

University of Dundee

Dundee, DD1 4HJ

United Kingdom

Author contribution statement:

NS & JB conceived the study. JB, JK, RR, LR and NS led the design of the research. JB, NS and RR drafted the manuscript. JB, RR, JK, LR, GA, CB, AC, RC, OFF, JH, NL, JvO, MP, KS, EU, RV, JW, KW, GZ, NS made substantial contributions to the conception or design of the work. JB, RR, JK, LR, GA, CB, AC, RC, OFF, JH, NL, JvO, MP, KS, EU, RV, JW, KW, GZ, NS revised it critically for important intellectual content. JB, RR, JK, LR, GA, CB, AC, RC, OFF, JH, NL, JvO, MP, KS, EU, RV, JW, KW, GZ, NS gave their final approval of the version to be published. JB, RR, JK, LR, GA, CB, AC, RC, OFF, JH, NL, JvO, MP, KS, EU, RV, JW, KW, GZ, NS agree to be accountable for all aspects of the work in ensuring that questions related to the accuracy or integrity of any part of the work are appropriately investigated and resolved.

Declaration of funding and other support:

For peer review only - http://bmjopen.bmj.com/site/about/guidelines.xhtml

This research is supported in part by the National Institute for Health Research (NIHR) using Official Development Assistance (ODA) funding (Grant: 17/63/130: NIHR Global Health Research Group: Improving Outcomes in Mental and Physical Multimorbidity and Developing Research Capacity (IMPACT) in South Asia at the University of York).

The research is also receiving support from Bradford District Care NHS Foundation Trust. Oscar Flores-Flores is supported by the Research Training in Chronic, non-communicable respiratory diseases in Peru, Fogarty International Center, US National Institutes of Health (D43TW011502).

The core outcome set development is also supported by the Global Alliance for Chronic Diseases (GACD) Multimorbidity Working Group; the World Psychiatry Association; and by Cochrane Common Mental Disorders which is funded by NIHR (NIHR 129457) and Cochrane Global Mental Health (https://globalmentalhealth.cochrane.org). It is supported by the NCD Alliance by dissemination of participation opportunities to people living with multiple health conditions in its network.

Competing interests:

None declared.

Acknowledgements:

The authors thank Job van Boven (University Medical Center Groningen, University of Groningen, The Netherlands) and Meena Daivadanam (Department of Food Studies, Nutrition and Dietetics, Uppsala University, Sweden) for comments on earlier drafts of this protocol.

For peer review only

Abstract

Introduction

'Multimorbidity' describes the presence of two or more long-term conditions, which can include communicable, non-communicable diseases, and mental disorders. The rising global burden from multimorbidity is well-documented, but trial evidence for effective interventions in low- and middle-income countries (LMICs) is limited. Selection of appropriate outcomes is fundamental to trial design to ensure cross-study comparability, but there is currently no agreement on a core outcome set (COS) to include in trials investigating multimorbidity specifically in LMICs. Our aim is to develop international consensus on two COS for trials of interventions to prevent and treat multimorbidity in LMIC settings.

Methods and Analysis

Following methods recommended by the Core Outcome Measures in Effectiveness Trials initiative, the development of these two COS will occur together, in parallel in three stages: (1) generation of a long list of potential outcomes for inclusion; (2) two-round online Delphi surveys; and (3) consensus meetings. First, to generate an initial list of outcomes, we will conduct a systematic review of multimorbidity intervention and prevention trials and interviews with people living with multimorbidity and their caregivers in LMICs. Outcomes will be classified using an outcome taxonomy. Two-round Delphi surveys will be used to elicit importance scores for these outcomes from people living with multimorbidity, caregivers, healthcare professionals, policy makers, and researchers in LMICs. Finally, consensus meetings including all of these stakeholders will be held to agree outcomes for inclusion in the two COS.

Ethics and dissemination

The study has been approved by the Research Governance Committee of the Department of Health Sciences, University of York, UK [HSRGC/2020/409/D:COSMOS]. Each participating country/research group will obtain local ethics board approval. Informed consent will be obtained from all participants. We will disseminate findings through peer-reviewed open access publications, and presentations at global conferences selected to reach a wide range of LMIC stakeholders.

For peer review only - http://bmjopen.bmj.com/site/about/guidelines.xhtml

Trial registration:

PROSPERO ID CRD42020197293

COMET https://www.comet-initiative.org/Studies/Details/1580

Keywords: Core Outcome Set, Comorbidity, Multimorbidity, Low- and Middle-Income Countries, Delphi, Consensus

Strengths and limitations of this study

- The development process follows guidelines and best practice recommendations for developing core outcome sets and integrates four sources of information.
- Interviews with people living with multimorbidity and caregivers are conducted in several LMICs (in South Asia, Africa and Latin America), by local teams, and in local languages to identify outcomes relevant to them.
- The Delphi survey and consensus meetings are conducted in English which limits the breadth of participation in these stages of the process.
- Despite involvement of a wide range of LMIC stakeholders in the process, there may be some limitations to the generalisability of the final core outcome sets due to the heterogeneity of target conditions and the diversity of countries, cultures and experiences.

For peer review only - http://bmjopen.bmj.com/site/about/guidelines.xhtml

INTRODUCTION

The rising global burden from chronic diseases is widely recognised and its detrimental impact on individuals as well as on societies is well-documented(1, 2). Chronic diseases often co-occur in individuals and this 'multimorbidity' is associated with more frequent health care consultations(3), longer hospital stays(4), worse health-related quality of life(5-7), increased health care costs(8), higher out-of-pocket expenditure for health care(8), and higher mortality(9). These factors make multimorbidity a pressing challenge for health care systems as well as for patients, families and caregivers(2, 8, 10). We define multimorbidity as the presence of two or more chronic (or long term) health conditions(11) encompassing communicable and non-communicable diseases and mental disorders, e.g. depression and diabetes occurring together. Chronic communicable diseases are those that affect people over a number of months or years e.g. HIV or tuberculosis. Non-communicable diseases are chronic conditions that cannot be directly transmitted between people, e.g. diabetes, heart disease and chronic lung disease.

Estimates of the prevalence of multimorbidity in high-income countries (HIC) vary from 25% to 60% in health care and community settings(12-14). The prevalence of multimorbidity in low- and middle-income countries (LMICs) appears to be lower but is set to rise rapidly due to demographic and lifestyle changes (11, 15, 16). A Cochrane review on the effectiveness of health service or patient-oriented interventions to improve outcomes in people with multimorbidity in primary care and community settings found most evidence was from HICs(17). This evidence cannot readily be applied to LMICs where differing patterns of disease, health care systems, resources and cultural considerations will affect the acceptability and effectiveness of interventions(11, 18). Trials of interventions to tackle multimorbidity specifically in LMICs are urgently needed(10).

To generate a robust evidence-base, results from individual trials will need to be compared and synthesised. This requires studies to use a 'core outcome set' (COS), i.e. an agreed minimum set of outcomes to be measured in all trials of a specific intervention for a health condition(19). A COS for multimorbidity research has been previously developed(20); it includes three outcomes: health-related quality of life, mental health, and mortality. However, a limitation of this COS is that it was largely based on the evidence and expert opinions from HICs, with no involvement of LMIC stakeholders in reaching consensus. This is a major barrier to its adoption in LMIC research, where there may be differing priorities for treatments amongst patients, families, health professionals and researchers, and therefore a different set of core outcomes may be required, specifically for LMIC settings(21, 22). A recent review discussed outcome measures across eight domains that may be suitable for multimorbidity research in LMICs(10). However, it did not systematically review the literature, patients and caregivers were not involved, and outcomes were not prioritised using

For peer review only - http://bmjopen.bmj.com/site/about/guidelines.xhtml

established consensus methods. The authors concluded that development of a COS for LMICs would be an important contribution to the field.

Whilst some research outcomes may be applicable to both LMICs and HICs, there are important differences in disease patterns and health care systems that need to be considered. For example, differences in severity of disease symptoms or complications and in healthcare availability and financing may influence outcome priorities(1, 15, 16, 21). In a recent trials methodology research priority setting exercise for LMIC settings, the choice of outcomes to measure was identified as the number one most important topic (23). This protocol outlines a plan to bridge the gap and develop two COS for research on the prevention and treatment of multimorbidity in an LMIC setting, using the minimum standards approach for developing COS(24). Separate COS are needed given the often differing targets for prevention and treatment interventions.

Aims and objectives

We aim to develop two COS for trials of interventions designed to (1) prevent and (2) treat multimorbidity in LMIC settings. The scope includes all types of interventions for multimorbidity – pharmacological, non-pharmacological, simple and complex in adults (aged 18 years and over) at risk of, or living with multimorbidity, in community, primary care and hospital settings in LMICs. Complex interventions are those with multiple interacting components (e.g., health risk behaviour change interventions), which contrast with 'simple' interventions, such as pharmacological interventions(25).

METHODS

We will follow approaches recommended by the Core Outcome Measures in Effectiveness Trials (COMET) initiative(26) and Outcome Measures in Rheumatoid Arthritis Clinical Trials (OMERACT(27)). The COS development has been registered with COMET (https://www.comet-initiative.org/Studies/Details/1580). The process will follow the minimum standards for the design of a COS study (COS-STAD(24)), which includes the careful consideration of the scope, stakeholders, and the consensus process. This protocol is structured according to the COS-STAP statement(28).

Our research team has broad global LMIC representation as well as experience of working in LMICs. The Core Outcome Set for Multimorbidity Studies (COSMOS) working group supporting the study draws on a network of 38 research teams in LMICs that will be actively involved in different stages of COS development. We will ensure patient, community and public involvement

For peer review only - http://bmjopen.bmj.com/site/about/guidelines.xhtml

throughout the development and delivery of the project, and include people with lived experience and their caregivers as members in the core research team.

COS development will involve three stages: (1) generation of a long list of outcomes, (2) two-round online Delphi consensus-building surveys and (3) online consensus meetings.

1. Outcome list generation

A long-list of outcomes will be generated from two sources: first, a systematic review of outcomes reported in trials of interventions conducted in LMICs for the prevention and treatment of multimorbidity, and second, using interviews with people living with multimorbidity and their caregivers from LMICs.

(1A) Systematic review

We will conduct a systematic review to identify an initial list of potential outcomes from trials of multimorbidity interventions conducted in adults living in LMICs. The systematic review protocol has been registered, and full details are available on PROSPERO (CRD42020197293).

Search methods:

We will search electronic databases (e.g. MEDLINE, Embase, PsycINFO) from 1990 to present. We will also search for relevant systematic reviews in The Cochrane Library, the Database of Abstracts of Reviews of Effects (DARE), PROSPERO and Joanna Briggs; and unpublished ongoing trials in ClinicalTrials.gov, ISRCTN registry and in the International Clinical Trials Registry Platform (http://apps.who.int/trialsearch). We will apply a study design filter to identify interventional studies. We will not limit inclusion by language.

Selection criteria

We will include randomised (individual, cluster, and cross-over randomisation) intervention studies published in either protocols or definitive reports. Studies of adults aged 18 years and above, living in LMICs, with multimorbidity, or at risk of multimorbidity will be eligible. LMICs will be defined using the 2019 World Bank definitions(29). Studies of all interventions, whether pharmacological, non-pharmacological, simple or complex, for the prevention or treatment of multimorbidity (or both) and any comparators will be included.

Study selection

Two reviewers will independently screen titles/ abstracts with any discrepancies resolved through consensus or if needed, by a third reviewer. Full texts will also be reviewed following a similar process.

For peer review only

For peer review only - http://bmjopen.bmj.com/site/about/guidelines.xhtml

Data collection

Data will be extracted from eligible studies on publication date, years when the trial was conducted, country, clinical setting (e.g., primary care, specialised services), type of intervention, target conditions, and outcomes, by a single reviewer, with 10% of extraction records also reviewed by a second senior reviewer.

Quality assessment

Risk of bias will not be assessed, as the purpose is only to assemble a list of outcomes that have been used in previous studies.

Data synthesis

Outcomes will be grouped based on whether they were used to evaluate prevention or treatment of multimorbidity. For both groups of outcomes, we will construct matrices to identify the outcomes, whether primary or secondary, their definitions and measures used in each study. We will tabulate the proportion of included studies that report on each outcome (including primary versus other outcome) and rank-order the outcomes accordingly. In a second step, to organise the outcomes we will use an outcome taxonomy.

The outcomes will be presented by intervention type (prevention, treatment), by combination of conditions that the trial targeted, and by region of the world (World Bank regions of LMICs(29)) in which the study was conducted.

(1B) Interviews

To identify outcomes of importance to people from LMICs living with multimorbidity and their families or caregivers, we will conduct semi-structured interviews. Participants will be approached by local research teams either in-person or via phone, post or on-line (based on the local research site resources and COVID-19 restrictions).

Inclusion criteria for interviews comprise: people living with (or caring for someone living with) two or more health conditions; at least 18 years of age; being able to visit the local partner organisation's site or access to telephone/Internet; and consenting to participate. Participants will be identified through contact with relevant health care services, support and advocacy groups, charities and use of social media including Twitter, adverts placed on public and patient involvement websites, snowballing techniques, and personal contacts. We will use purposive sampling(30) based on the following characteristics: age (over/under 65 years); sex (male/female); using community or primary care versus secondary/specialist healthcare services; and region (World Bank income groups: low, lower middle, upper middle(29)). We will include a range of LMICs in diverse geographic locations. We anticipate 15 to 20 participants from each group (45-

For peer review only - http://bmjopen.bmj.com/site/about/guidelines.xhtml

For peer review only

60 interviews), which should be sufficient to achieve diversity of viewpoints and data saturation(31).

We will collect demographic information (age, sex, marital status, highest level of education, socioeconomic status, occupation and disability. We have developed a semi-structured interview guide (see Appendix 1(26)), which will be translated and back-translated by local teams in the local language. Interviews will be conducted by researchers with experience of conducting qualitative interviews. All interviews will be audio recorded or if not possible (because of technology limitations or the participant withholding consent) will be recorded in contemporaneous notes.

The local research teams will be asked to transcribe and translate into English two sections of the interview, (i) participants' consent and (ii) content relevant to study outcomes. Interview transcripts will be reviewed to confirm health outcomes to put forward for the Delphi.

Outcomes identified in the interviews will be integrated with findings from the systematic review. All unique outcomes identified from the systematic review and interviews will be categorised into domains for presentation in the Delphi survey using Dodd's outcome taxonomy(32).

2. Delphi surveys with stakeholders

We will conduct separate Delphi surveys for the prevention and treatment COS; however, survey participants will be common to both.

We will recruit a range of stakeholders living or working in LMICs including the following groups: i) people living with multimorbidity and their family caregivers, ii) healthcare professionals experienced in working with patients with multimorbidity; iii) policy makers and managers; and iv) researchers interested in multimorbidity with experience in LMIC contexts. Participants will be identified using connected networks (e.g. NIHR Global Health Research Groups/Units, Global Alliance for Chronic Diseases, World Psychiatric Association, NCD Alliance) and our project network across 38 countries to distribute information about the study, including professional societies and non-government organisations relevant to multimorbidity and government ministries. For recruitment to the researcher stakeholder group, we will send personalised emails to corresponding authors living or working in LMICs identified via our systematic review and request them to snowball to co-authors and other relevant contacts.

We will aim to maintain a minimum of approximately 20 participants representing each stakeholder group (total approximately 80 participants) throughout Delphi rounds, with representation across the World Bank LMIC income groups(29). We will oversample for the first round based on an estimated attrition of 30% across rounds. In both rounds we will send three reminders across three

For peer review only - http://bmjopen.bmj.com/site/about/guidelines.xhtml

weeks to participants to complete the surveys and keep the surveys open for longer if needed to reduce attrition and improve the response rate.

(2A) Round one

We will include all outcomes identified through our systematic review and interviews, seeking advice from our community and public representatives to describe outcomes in lay terms. We will ensure the outcomes that enter the Delphi are non-disease specific (important for the scope of this COS), have relevance to LMIC, and avoid duplication. We will use an online survey tool (DelphiManager) to administer the surveys. We will pilot the questionnaire with eight individuals (two from each stakeholder group) to assess content validity and understanding.

We will list outcomes in domains according to the taxonomy classification. Since we are interested in assessing the importance of each outcome, we will ask participants to score each outcome (separately for prevention and for treatment) on a scale from 1 to 9 (1 to 3 = not important for inclusion; 4 to 6 = important but not critical; 7 to 9 = critical for inclusion(33)), considering relevance for and feasibility of the outcome in LMIC settings. There will also be an option for 'unable to score'. We will provide the opportunity for participants to add additional outcomes and other comments. We will collect demographic information to describe our study sample (Table 1). All responses, including from partially completed questionnaires will be used in analyses.

INSERT TABLE 1 ABOUT HERE

(2B) Round two

The research team will review any additional outcomes suggested in round one for inclusion. Any newly suggested outcomes will be taken forward if they are (i) relevant to the scope of the COS and (ii) not yet represented in the list of outcomes. All outcomes from round 1 and any new outcomes will be included in round 2.

In round two, we will provide participants with their own round one response; summarised responses of the whole group; and responses summarised by each stakeholder group. We will ask them to re-score the importance of each outcome in light of the extra information received, again

For peer review only - http://bmjopen.bmj.com/site/about/guidelines.xhtml

separately for prevention and treatment. New outcomes from round one will be provided for scoring on the same 1 to 9 importance scale.

We will summarise round 2 responses separately for prevention and treatment using descriptive statistics, noting for each outcome the number and percentage of responses in each of the following categories:

- 1 to 3 = not important for inclusion
- 4 to 6 = important but not critical
- 7 to 9 = critical for inclusion

We will categorise outcomes according to the predefined consensus definition described in Table 2(34-36). All responses, including from partially completed questionnaires will be used in analyses.

We will also examine the percentage distribution of scores for each outcome by stakeholder group, geographic region and World Bank income group and present these graphically for the consensus meeting.

At the end of the second round of Delphi surveys participants will be sent information about the consensus meeting and requested to contact the team if they are interested in participating.

3. Outcome consensus meetings

Due to the wide geographic distribution of participants, the final step in the consensus process will be online consensus meetings to agree (i) the COS for prevention of multimorbidity and (ii) the COS for treatment of multimorbidity.

Participants of the Delphi surveys who complete both rounds and who express an interest in attending the consensus meeting will be purposively selected to ensure similar numbers from each stakeholder group, geographical spread and representation from target World Bank income groups. The meetings will be chaired by a facilitator, experienced in COS development and will use a range of functions available in online meetings, such as breakout rooms for smaller group discussions, polls and 'chat' to facilitate meaningful participation. Results of the two-round Delphi surveys will be presented. Outcomes that have reached consensus 'in' or 'no consensus' (see Table 2) will be discussed further.

We will use a modified nominal group technique to achieve consensus. This will involve iterative small and whole group discussions and ranking of outcomes in order to agree which should be included in the two COS. To avoid duplication or overlap, the selected outcomes will also be discussed to ensure each relates to a distinct construct. If consensus cannot be reached on all

For peer review only - http://bmjopen.bmj.com/site/about/guidelines.xhtml

outcomes or there are outstanding issues to consider, we will convene separate meetings with the stakeholder members until a final COS is ratified.

For people living with multimorbidity and their caregivers, we will provide additional support before the consensus meetings to explain the process and enable them to participate more fully in the discussions and ranking.

INSERT TABLE 2 ABOUT HERE

PATIENT AND PUBLIC INVOLVEMENT

There were no people living with the condition nor their caregivers involved in the planning of the study. Both will be involved in the project management group overseeing the Delphi surveys and consensus meeting. Both will participate in both Delphi surveys and consensus meeting.

ETHICS

The Ethics committee of the University of York has approved the study [HSRGC/2020/409/D: COSMOS]. Approvals will also be sought from relevant local ethics committees for all participating sites.

We will obtain written consent from participants in interviews and consensus meetings (or where conducted online, verbal consent will be recorded). For the Delphi surveys, we will seek consent for participation as part of the online questionnaire and consent to store contact data for completion of the next Delphi round and for invitation to consensus meetings. We will emphasise that participation is voluntary, participants are free to withdraw at any stage, and survey responses will be anonymized.

Data management

Interview data as well as anonymized Delphi data after secure download will be stored on a secure and encrypted database on a secure password protected server (the University of York, Department of Health Sciences File store Area). The access, use and storage of sensitive or confidential data will be conducted in accordance with the University of York Data Security Policy and Handling Sensitive Data Guidance.

For peer review only

For peer review only - http://bmjopen.bmj.com/site/about/guidelines.xhtml

DISSEMINATION

We will disseminate our findings through peer-reviewed and open access publications, and presentations at global conferences, selected to reach a wide range of LMIC stakeholders including health professionals and researchers and taking into account geographic locations. We will also provide lay summaries including infographics in relevant languages for community and public audiences and share these with interview and Delphi participants. Such summaries and short briefing papers will be made available freely on the COSMOS web page (https://www.impactsouthasia.com/) and in formats which are at the moment being successfully developed and trialled in the associated research networks (Global Alliance for Chronic Diseases website, World Psychiatric Association). In the longer term, success of dissemination will be assessed by how frequently the Multimorbidity COS is used in trials and cited in manuscripts(37).

DISCUSSION

We aim to develop and report two COS for multimorbidity outcomes specifically for LMICs by identifying outcomes that have been reported in the literature, including the views of people living with multimorbidity and their caregivers through interviews, and developing consensus using Delphi surveys and consensus meetings. Development of the two COS aims to increase the consistency of selection, collection and reporting of outcomes in future studies of interventions to prevent and to treat multimorbidity.

A considerable strength of the study is the involvement of people living with multimorbidity and their family caregivers throughout, including in interviews to gain their perspective on important outcomes. This involvement is increasingly recognised as an important contributor to COS(24, 38, 39) as it highlights new outcomes from the service user perspective. Aiming for approximately fifty participants from interviews and a wide range of participants in the Delphi globally(39), the study will generate outcomes and gain consensus from a range of diverse settings.

The research team comprises experienced researchers and experts in the field of multimorbidity and COS development. The support of the Global Alliance for Chronic Disease (GACD), the World Psychiatry Association (WPA) and partners in IMPACT (Improving Outcomes in Mental and Physical Multimorbidity and Developing Research Capacity) will help provide links to local groups and identify appropriate stakeholders for the consensus process as well as for the dissemination of the outcomes generated from the study.

There are some relevant limitations. First, the scope is broad; despite the systematic effort to conduct a comprehensive and inclusive COS development, with involvement of a wide range of LMIC stakeholders, it is possible that there may be limitations to the generalisability of the final

For peer review only - http://bmjopen.bmj.com/site/about/guidelines.xhtml

core outcome sets due to heterogeneity of interventions, target conditions and diversity of countries, cultures and experiences.

A further limitation is that although the study aims to develop COS that will be relevant to LMICs more generally, it will only include representation from some LMIC regions. We recognise that LMICs are not a homogenous category and social and cultural differences will necessitate future research investigating how these COS can be applied to other regions. Nevertheless, we included literature from all LMICs in generating the list of outcomes, and the diverse range of countries represented means it is likely that the outcomes selected will be those common across countries and cultures. We do not therefore intend to define the regions for application, other than saying these COS are for LMIC settings.

To be flexible and adaptable for a range of LMIC settings, and feasible within the available time and resource constraints, the planned interviews are limited in number and relatively short and semi-structured and the transcription will focus only on the most relevant sections. Due to internationally different clinical standards and the potential stigma associated with labelling people as "at risk", we decided not to conduct interviews focusing on prevention only. This may risk missing outcomes which would be obtained from a more in-depth exploration.

Although our aim is to secure participation from a wide spread of geographical areas, there are a number of challenges to involvement. To take part in Delphi surveys and consensus meetings, English proficiency, a computer and internet access will be required. This poses challenges for engagement with some stakeholder groups, inclusiveness and participant attrition. Additionally, since patient and public involvement is still a developing concept in many contexts and specifically in global health research(40-43) identifying appropriate patient representatives for our consensus panel may prove challenging.

Despite these challenges, this is the first attempt to develop core outcome sets for multimorbidity seeking input from a wide range of LMIC partners and participants(21). Additionally, the involvement of various experts in COS and multimorbidity strengthens the COS development process.

Our COSMOS study follows a systematic widely practiced approach to generate core outcomes and meets the methodological standards put forth by the COMET initiative. It will generate a template for steps in developing international consensus involving LMICs, and two COS for use in future multimorbidity prevention and treatment trials, helping to generate evidence to address this important global health priority.

For peer review only - http://bmjopen.bmj.com/site/about/guidelines.xhtml

References

1. Murray CJL, Barber RM, Foreman KJ, Ozgoren AA, Abd-Allah F, Abera SF, et al. Global, regional, and national disability-adjusted life years (DALYs) for 306 diseases and injuries and healthy life expectancy (HALE) for 188 countries, 1990–2013: quantifying the epidemiological transition. The Lancet. 2015;386(10009):2145-91.

2. Pati S, Swain S, Knottnerus JA, Metsemakers JFM, van den Akker M. Magnitude and determinants of multimorbidity and health care utilization among patients attending public versus private primary care: a cross-sectional study from Odisha, India. Int J Equity Health. 2020;19(1):57.

3. Palladino R, Tayu Lee J, Ashworth M, Triassi M, Millett C. Associations between multimorbidity, healthcare utilisation and health status: evidence from 16 European countries. Age Ageing. 2016;45(3):431-5.

4. Mai Q, Holman CD, Sanfilippo FM, Emery JD. The impact of mental illness on potentially preventable hospitalisations: a population-based cohort study. BMC Psychiatry. 2011;11:163.

5. Barra M, Augestad LA, Whitehurst DG, Rand-Hendriksen K. Examining the relationship between health-related quality of life and increasing numbers of diagnoses. Qual Life Res. 2015;24(12):2823-32.

6. Ou HT, Lin CY, Erickson SR, Balkrishnan R. Refined comorbidity index based on dimensionality of comorbidity for use in studies of health-related quality of life. Qual Life Res. 2016;25(10):2543-57.

7. Tyack Z, Frakes KA, Barnett A, Cornwell P, Kuys S, McPhail S. Predictors of health-related quality of life in people with a complex chronic disease including multimorbidity: a longitudinal cohort study. Qual Life Res. 2016;25(10):2579-92.

8. Zhao Y, Atun R, Oldenburg B, McPake B, Tang S, Mercer SW, et al. Physical multimorbidity, health service use, and catastrophic health expenditure by socioeconomic groups in China: an analysis of population-based panel data. The Lancet Global Health. 2020;8(6):e840-e9.

9. Gonzalez R, Lopez-Herce J. Mortality of patients with chronic disease: an increasing problem. J Pediatr (Rio J). 2019;95(6):625-7.

10. Hurst JR, Agarwal G, van Boven JFM, Daivadanam M, Gould GS, Wan-Chun Huang E, et al. Critical review of multimorbidity outcome measures suitable for low-income and middle-income country settings: perspectives from the Global Alliance for Chronic Diseases (GACD) researchers. BMJ Open. 2020;10(9).

11. The Academy of Medical Sciences. Multimorbidity: a priority for global health research [Internet]. 2018 Apr [cited 2021 Jan 7] p. 127. Available from: https://acmedsci.ac.uk/file-download/82222577

12. Brett T, Arnold-Reed DE, Popescu A, Soliman B, Bulsara MK, Fine H, et al. Multimorbidity in patients attending 2 Australian primary care practices. Ann Fam Med. 2013;11(6):535-42.

For peer review only

For peer review only - http://bmjopen.bmj.com/site/about/guidelines.xhtml

13. Marengoni A, Winblad B, Karp A, Fratiglioni L. Prevalence of chronic diseases and multimorbidity among the elderly population in Sweden. Am J Public Health. 2008;98(7):1198-200.

14. Schafer I, Hansen H, Schon G, Hofels S, Altiner A, Dahlhaus A, et al. The influence of age, gender and socio-economic status on multimorbidity patterns in primary care. First results from the multicare cohort study. BMC Health Serv Res. 2012;12:89.

15. Sara Afshar, Paul J. Roderick, Paul Kowal, Borislav D. Dimitrov, Hill AG. Global Patterns of Multimorbidity: A Comparison of 28 Countries Using the World Health Surveys. In: M. Nazrul Hoque, Beverly Pecotte, McGehee MA, editors. Applied Demography and Public Health in the 21st Century. Switzerland: Springer International Publishing 2017. p. 381-402.

16. Xu X, Mishra GD, Jones M. Mapping the global research landscape and knowledge gaps on multimorbidity: a bibliometric study. J Glob Health. 2017;7(1):010414.

17. Smith SM, Wallace E, O'Dowd T, Fortin M. Interventions for improving outcomes in patients with multimorbidity in primary care and community settings. Cochrane Database Syst Rev. 2016;3:CD006560.

18. Pati S, Swain S, Hussain MA, van den Akker M, Metsemakers J, Knottnerus JA, et al. Prevalence and outcomes of multimorbidity in South Asia: a systematic review. BMJ Open. 2015;5(10):e007235.

19. Prinsen CAC, Vohra S, Rose MR, Boers M, Tugwell P, Clarke M, et al. How to select outcome measurement instruments for outcomes included in a "Core Outcome Set" – a practical guideline. Trials. 2016;17(1):449.

20. Smith SM, Wallace E, Salisbury C, Sasseville M, Bayliss E, Fortin M. A Core Outcome Set for Multimorbidity Research (COSmm). The Annals of Family Medicine. 2018;16(2):132-8.

21. Lee A, Davies A, Young AE. Systematic review of international Delphi surveys for core outcome set development: representation of international patients. BMJ Open. 2020;10(11):e040223.

22. Hailemariam M, Pathare S. The missing global in global mental health. The Lancet Psychiatry. 2020;7(12):1011-2.

23. Rosala-Hallas A, Bhangu A, Blazeby J, Bowman L, Clarke M, Lang T, et al. Global health trials methodological research agenda: results from a priority setting exercise. Trials. 2018;19(1):48.

24. Kirkham JJ, Davis K, Altman DG, Blazeby JM, Clarke M, Tunis S, et al. Core Outcome Set-STAndards for Development: The COS-STAD recommendations. PLoS Med. 2017;14(11):e1002447.

25. Petticrew M. When are complex interventions 'complex'? When are simple interventions 'simple'? Eur J Public Health. 2011;21(4):397-8.

26. Williamson PR, Altman DG, Bagley H, Barnes KL, Blazeby JM, Brookes ST, et al. The COMET Handbook: version 1.0. Trials. 2017;18(Suppl 3):280.

27. OMERACT Handbook [Internet]. OMERACT Handbook. 2018 [cited 2021 Jan 7]. Available from: https://omeracthandbook.org/

For peer review only - http://bmjopen.bmj.com/site/about/guidelines.xhtml

28. Kirkham JJ, Gorst S, Altman DG, Blazeby JM, Clarke M, Tunis S, et al. Core Outcome Set-STAndardised Protocol Items: the COS-STAP Statement. Trials. 2019;20(1):116.

29. World Bank Country and Lending Groups [Internet]. The World Bank. [cited 2021 Jan 7]. Available from: https://datahelpdesk.worldbank.org/knowledgebase/articles/906519-world-bank-country-and-lending-groups

30. Pope C, Mays N. Reaching the parts other methods cannot reach: an introduction to qualitative methods in health and health services research. BMJ. 1995;311(6996):42-5.

31. Francis JJ, Johnston M, Robertson C, Glidewell L, Entwistle V, Eccles MP, et al. What is an adequate sample size? Operationalising data saturation for theory-based interview studies. Psychol Health. 2010;25(10):1229-45.

32. Dodd S, Clarke M, Becker L, Mavergames C, Fish R, Williamson PR. A taxonomy has been developed for outcomes in medical research to help improve knowledge discovery. J Clin Epidemiol. 2018;96:84-92.

33. Guyatt GH, Oxman AD, Kunz R, Atkins D, Brozek J, Vist G, et al. GRADE guidelines: 2. Framing the question and deciding on important outcomes. J Clin Epidemiol. 2011;64(4):395-400.

34. Harman NL, Wilding JPH, Curry D, Harris J, Logue J, Pemberton RJ, et al. Selecting Core Outcomes for Randomised Effectiveness trials In Type 2 diabetes (SCORE-IT): a patient and healthcare professional consensus on a core outcome set for type 2 diabetes. BMJ Open Diabetes Res Care. 2019;7(1):e000700.

35. Quirke FA, Healy P, Bhraonain EN, Daly M, Biesty L, Hurley T, et al. COHESION: core outcomes in neonatal encephalopathy (protocol). Trials. 2021;22(1):125.

36. Williamson PR, Altman DG, Blazeby JM, Clarke M, Devane D, Gargon E, et al. Developing core outcome sets for clinical trials: issues to consider. Trials. 2012;13(1):132.

37. Hughes KL, Clarke M, Williamson PR. A systematic review finds Core Outcome Set uptake varies widely across different areas of health. J Clin Epidemiol. 2021;129:114-23.

38. Jones JE, Jones LL, Keeley TJ, Calvert MJ, Mathers J. A review of patient and carer participation and the use of qualitative research in the development of core outcome sets. PLoS One. 2017;12(3):e0172937.

39. Young B, Bagley H. Including patients in core outcome set development: issues to consider based on three workshops with around 100 international delegates. Res Involv Engagem. 2016;2:25.

40. Bedwell C, Lavender T. Giving patients a voice: implementing patient and public involvement to strengthen research in sub-Saharan Africa. J Epidemiol Community Health. 2020;74(4):307-10.

41. Cook N, Siddiqi N, Twiddy M, Kenyon R. Patient and public involvement in health research in low and middle-income countries: a systematic review. BMJ Open. 2019;9(5):e026514.

42. Simkhada B, van Teijlingen E, Naheed A, Warren A, Green S. Importance of involving patients and public in health research in Bangladesh and Nepal. Int J Technol Assess Health Care. 2020;37:e10.

For peer review only

For peer review only - http://bmjopen.bmj.com/site/about/guidelines.xhtml

577 43. Beran D, Pesantes MA, Berghusen MC, Hennig BJ, Jacobi J, Lazo-Porras M, et al.
578 Rethinking research processes to strengthen co-production in low and middle income
579 countries. BMJ. 2021;372:m4785.

580

581

For peer review only

Table 1: Delphi Stakeholder Characteristics

Stakeholder group	Characteristic
People with multimorbidity/family members/caregivers	
	Age (≤65; >65)
	Sex (male; female)
	Multimorbidities
	Community/primary care, secondary care; specialist healthcare
	Level of formal education (<= 10 years/>10years)
	Region of residence
Health professionals	
	Profession (doctor, nurse, allied health, community workers)
	Specialist area relevant to multimorbidity
	Region of residence
Policy makers and managers	
	Region of residence
Researchers	
	Specialist area relevant to multimorbidity
	Region of residence

For peer review only

For peer review only - http://bmjopen.bmj.com/site/about/guidelines.xhtml

Table 2: Definition of consensus for outcomes in the Delphi surveys

Consensus IN	Consensus that outcome should be included in the core outcome set	70% or more participants scoring 7–9<br>AND<br>< 15% participants scoring 1–3
Consensus OUT	Consensus that outcome should not be included in the core outcomes set	50% or fewer participants scoring 7 to 9 in each stakeholder group
No consensus	Uncertainty about importance of outcome	Anything else

For peer review only

Online Appendix to Boehnke et al., Development of a core outcome set for multimorbidity trials in low- and middle-income countries (COSMOS): Study Protocol. medRXix.

Appendix A: Interview guide

Development of a core outcome set for multimorbidity studies in low- and middle-income countries (COSMOS)

Interview Guide for Patients

Version: 1.3
Date: 28-10-20

For peer review only

Demographics (age, gender, marital status, highest level of education, socioeconomic status, occupation and disability should be recorded at eligibility checking/recruitment) need to be collected on a separate 'demographics' sheet.

Introduction:

Our goal is to understand what outcomes are important to you in preventing or treating co-existing chronic illnesses. We also want to identify what terms you might prefer to use to describe these outcomes.

What is an outcome?

To help patients, their family, doctors and other health professionals make decisions about treatments, we need evidence about what works best. Treatments are developed and tested by researchers to make sure they work and are safe. To do this, researchers need to look at the effects those treatments have on patients.

Researchers do this by measuring an 'outcome'. For example, in a study of how well a new asthma treatment works, 'outcomes' might include: a measure of how fast the asthma attack gets better; how many times a person has to stay in hospital because of asthma; or how well a person can get on with their usual activities because of asthma.

What is a core outcome set?

If all studies of a particular health condition(s) use the same outcomes, they can all be compared and combined. When a set of main outcomes has been agreed for a health condition(s), it's called a 'core outcome set'. If studies use these core outcomes, we could bring together all the studies to get a better understanding of which treatments are best.

What do we need you to tell us?

Core outcomes have to be relevant to patients, family members as well as health professionals and researchers. Some outcomes may be more important to patients and families than to healthcare professionals but without asking we will not know this and they might not get included in the core outcome list.

[Interviewer, before proceeding to the interview please check (if not already done):
- whether the participant information sheet had been shared and discussed;
- whether consent was obtained and documented.]

Interview:

As a quick reminder, we are interested in your experience of living with [living with someone living with] two or more health conditions at the same time. This is what we mean when using the terms "co-existing" or "multimorbidity". With health conditions we mean mental disorders, non-communicable diseases, and/or communicable diseases. In this interview we are interested in your view on what matters to you as the result of treatment and/or care for your conditions.

1. Please can you tell me how long (approximately) you [your family member] have had these co-existing chronic illnesses?

2. Where have you received treatment for these?
Probes: In the community, primary care, hospital, tertiary centre.

3. (For family members only) What is your relationship to your family member with these conditions?

4. Please tell me a little bit about your (your family member's) experience living with these conditions
Probe: What matters to you the most about the impact of these conditions?

5. What do you (your family member) hope to gain through treatment or care?

6. Speaking from your experience, how do you know that a treatment for your conditions is working?

[Interviewer, to finish off this question, ask]: What would you say your priorities are?

For peer review only - http://bmjopen.bmj.com/site/about/guidelines.xhtml

For peer review only

7. Considering this experience, what would you want future studies of prevention and treatment to look at in order to make it better for others in the future?

8. Is there anything else you'd like to talk about in relation to research for treatments to prevent or treat these conditions?

For peer review only

For peer review only - http://bmjopen.bmj.com/site/about/guidelines.xhtml

Core Outcome Set-STAndardised Protocol Items/COS-STAP Statement* accompanying Boehnke et al., Development of a core outcome set for multimorbidity trials in low- and middle-income countries (COSMOS): Study Protocol. medRXix.

*Kirkham, J.J., Gorst, S., Altman, D.G. *et al.* Core Outcome Set-STAndardised Protocol Items: the COS-STAP Statement. *Trials* **20,** 116 (2019). https://doi.org/10.1186/s13063-019-3230-x

TITLE/ABSTRACT			Referring to pages in manuscript submission without mark-up
Title	1a	Identify in the title that the paper describes the protocol for the planned development of a COS	page 1, lines 1-2
Abstract	1b	Provide a structured abstract	page 4, lines 84-111
INTRODUCTION			
Background and objectives	2a	Describe the background and explain the rationale for developing the COS, and identify the reasons why a COS is needed and the potential barriers to its implementation	pages 6-7, lines 137-180
	2b	Describe the specific objectives with reference to developing a COS	page 7, 181-188
Scope	3a	Describe the health condition(s) and population(s) that will be covered by the COS	page 7, 181-188
	3b	Describe the intervention(s) that will be covered by the COS	page 7, 181-188
	3c	Describe the context of use for which the COS is to be applied	page 7, 181-188
METHODS			
Stakeholders	4	Describe the stakeholder groups to be involved in the COS development process, the nature of and rationale for their involvement and also how the individuals will be identified; this should cover involvement both as members of the research team and as participants in the study	Interviews pages 9-10, lines 255-266;<br>Delphi pages 10-11, lines 282-298;<br>Consensus meeting page 12, 346-348.

For peer review only

Information sources	5a	Describe the information sources that will be used to identify the list of outcomes. Outline the methods or reference other protocols/papers	Systematic review pages 8-9. lines 211-231;<br>Interviews pages 9-10, lines 251-275;<br>First round of Delphi pages 11, lines 311-312
	5b	Describe how outcomes may be dropped/combined, with reasons	page 11, lines 300-305
Consensus process	6	Describe the plans for how the consensus process will be undertaken	pages 12-13, 343-362
Consensus definition	7a	Describe the consensus definition	pages 12-13, lines 354-359, and Table 2
	7b	Describe the procedure for determining how outcomes will be added/combined/dropped from consideration during the consensus process	pages 12-13, lines 351-359, and Table 2
ANALYSIS			
Outcome scoring/feedback	8	Describe how outcomes will be scored and summarised, describe how participants will receive feedback during the consensus process	page 12, lines 349-359
Missing data	9	Describe how missing data will be handled during the consensus process	page 11, line 313
ETHICS and DISSEMINATION			
Ethics approval/informed consent	10	Describe any plans for obtaining research ethics committee/institutional review board approval in relation to the consensus process and describe how informed consent will be obtained (if relevant)	page 13, lines 373-381
Dissemination	11	Describe any plans to communicate the results to study participants and COS users, inclusive of methods and timing of dissemination	page 14, lines 389-398
ADMINISTRATIVE INFORMATION			
Funders	12	Describe sources of funding, role of funders	pages 2-3, lines 58-61

For peer review only

Conflicts of interest	13	Describe any potential conflicts of interest within the study team and how they will be managed	page 3, line 73

For peer review only
